# Supplementary material for: Diamond Surfaces with Lateral Gradients for Systematic Optimization of Surface Chemistry for Relaxometry – a Low-Pressure Plasma-Based Approach
Source: Langmuir. 2024 Oct 18;40(43):23007–17. doi: 10.1021/acs.langmuir.4c03171 (PMC11526373; doi:10.1021/acs.langmuir.4c03171)
Supplement: Supplementary file 1 — la4c03171_si_001.pdf [file la4c03171_si_001.pdf]

Supplementary Material

Diamond surfaces with lateral gradients for systematic optimization of surface chemistry for relaxometry – A low pressure plasma-based approach

Yuchen Tian[#,1], Ari R. Ortiz Moreno[#,1], Mayeul Chipaux[1,2,*] Kaiqi Wu[1], Felipe P. Perona Martinez[1], Hoda Shirzad[2], Thamir Hamoh, Aldona Mzyk[1], Patrick van Rijn[1], Romana Schirhagl[1,*]

1 Groningen University, University Medical Center Groningen, Antonius Deusinglaan 1, 9713 AW Groningen, Netherlands,

2 Institute of Physics, École Polytechnique Fédérale de Lausanne (EPFL), CH-1015 Lausanne, Switzerland.

Corresponding author emails:

*romana.schirhagl@gmail.com

*mayeul.chipaux@epfl.ch

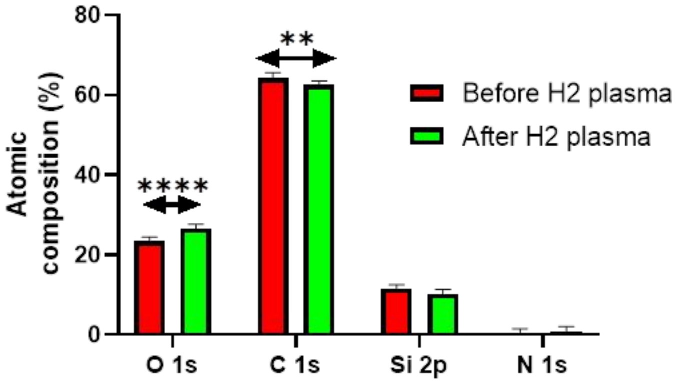

Figure S1: Changes in the atomic composition of the diamond surfaces due to hydrogen plasma treatment. For each group, the atomic composition was measured 3 times. Statistical significance was determined using an ANOVA *** $p < 0.001$, **** $p < 0.0001$. The slight increase in oxygen concentration might occur after the treatment itself when the sample is exposed to ambient air.

Table S1: Analysis of the C1s peak in XPS. Peak assignments are done based on [1,2]

**Diamond before plasma**	Position	Area	%	Assignment
	284,8	22957	13,5	SP2/CH
	286,53	145101	85,32	SP3
	288,38	1437	0,84	CO

	Position	Area	%	Assignment
	289,74	572	0,34	CO
**Air plasma (less exposed)**	Position	Area	%	Assignment
	284,8	12576	10	SP2/CH
	286,41	107015	85,08	SP3
	288,16	5078	4,04	CO
	289,95	1108	0,88	CO
**Air plasma (more exposed)**	Position	Area	%	Assignment
	284,8	19674	11,93	SP2/CH
	286,45	137074	83,11	SP3
	288,12	8175	4,96	CO
**H_2 plasma**	Position	Area	%	Assignment
	284,9707	8465,39	24,03	SP2/CH
	285,9386	24784,04	70,34	SP3
	287,1296	1984,59	5,633	CO

XPS data from air plasma treated samples

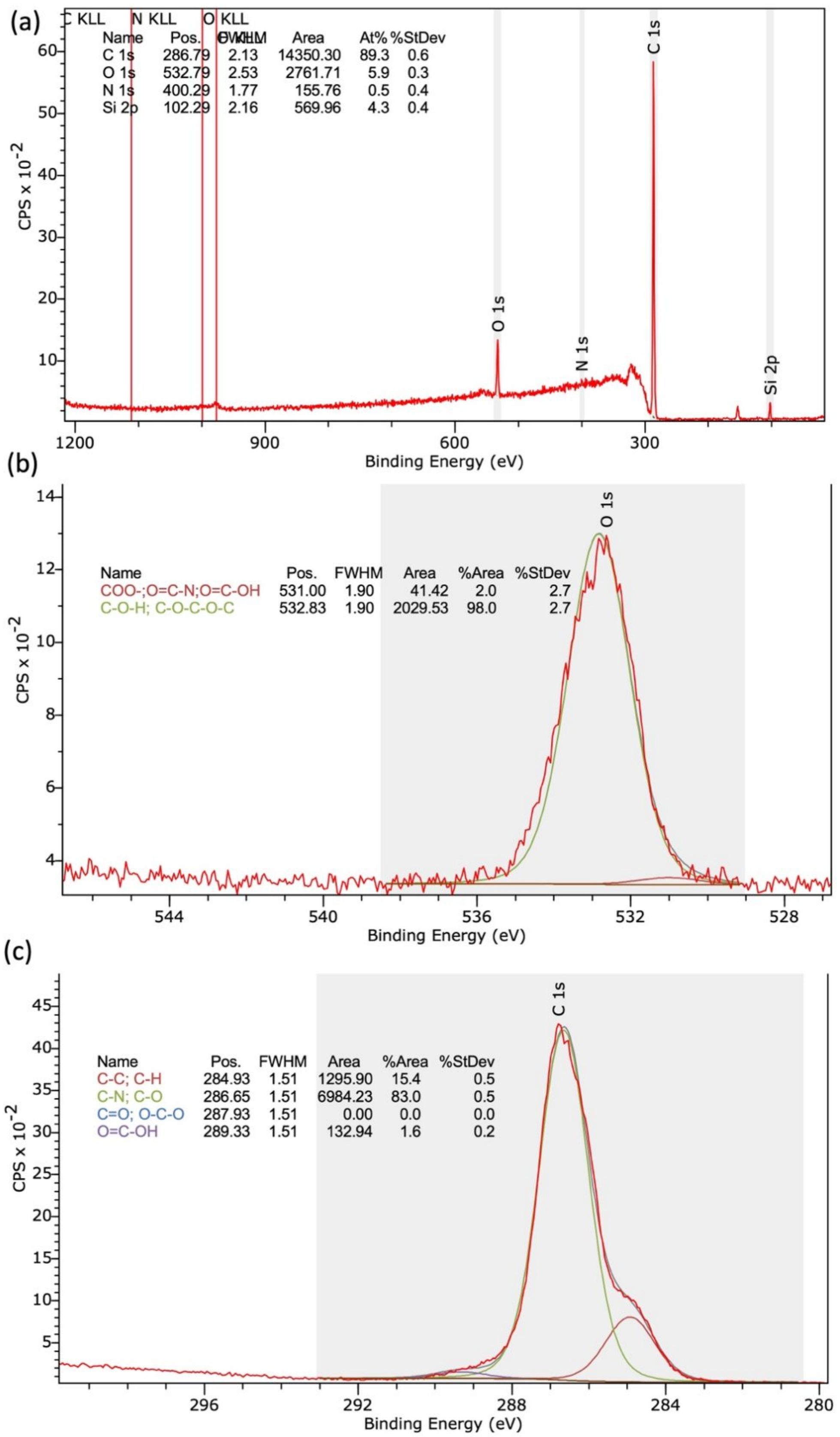

Figure S2. Characterisation of the diamond surface before plasma treatment (a) shows the entire XPS spectrum (b) an analysis of the oxygen group peak. (c) is the area of he C1s peak

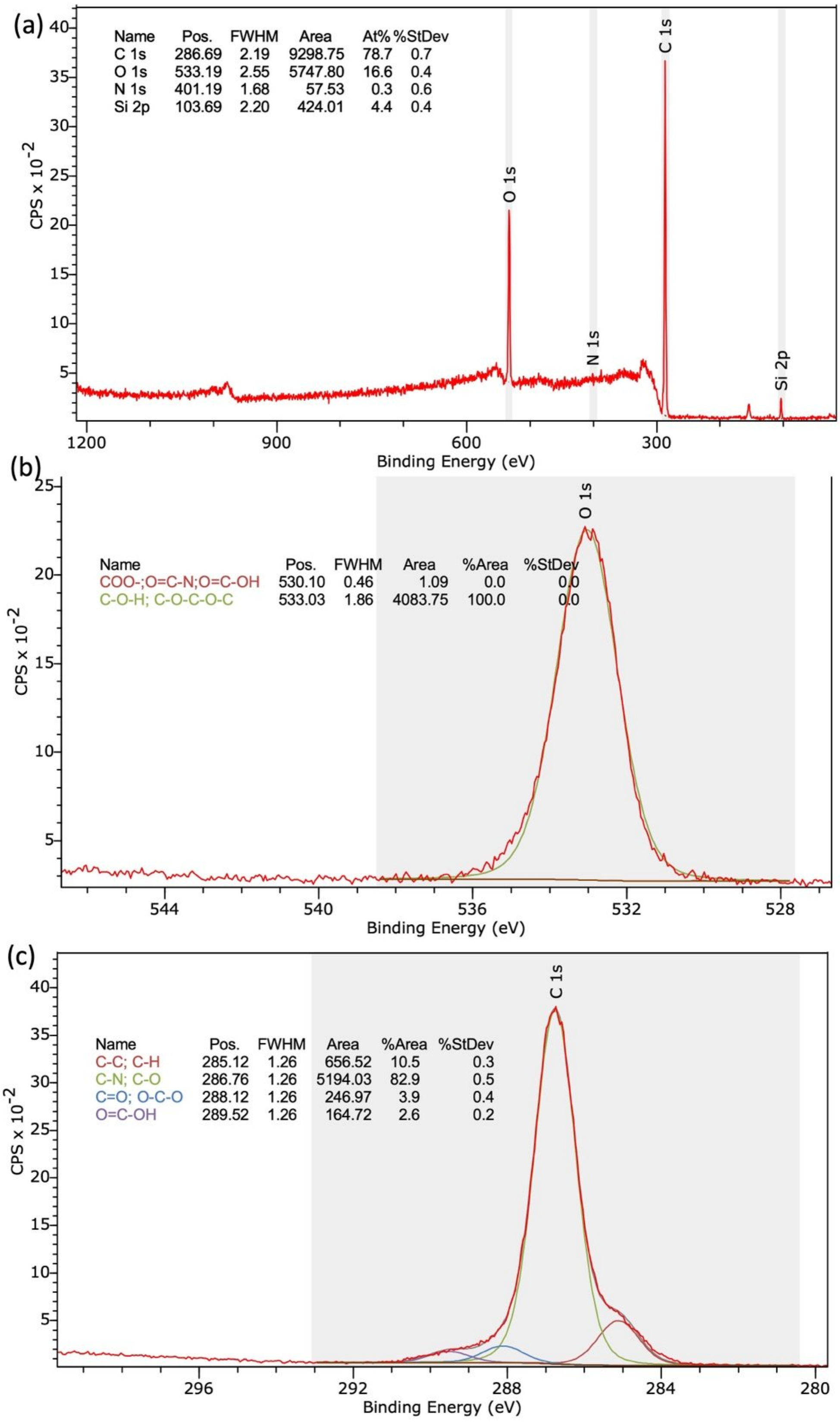

Figure S3. Characterisation of the chemical gradient across the diamond surface treated with air plasma: Position 1 (less exposed side) (a) shows the entire XPS spectrum (b) an analysis of the oxygen group peak. (c) is the area of the C1s peak

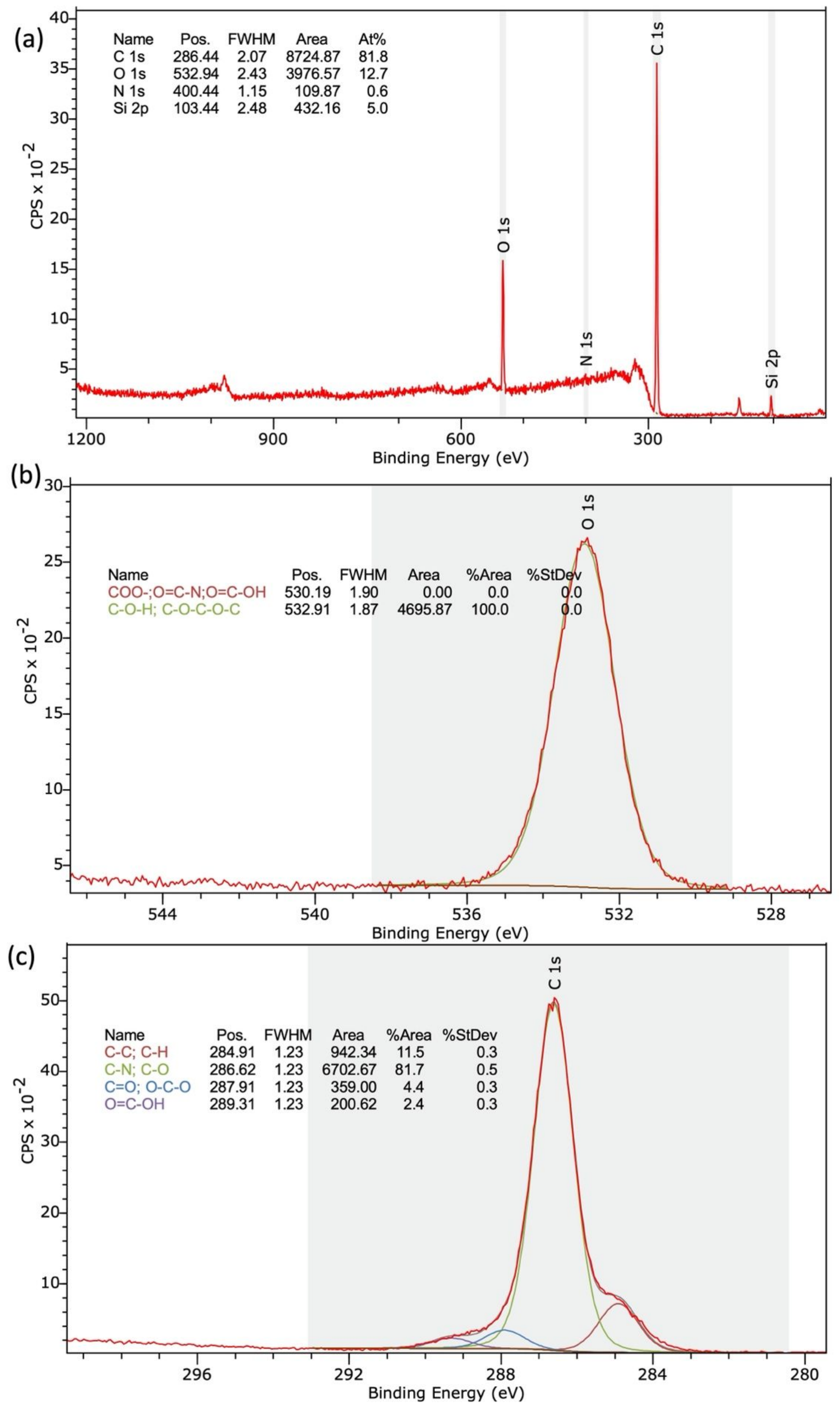

Figure S4. Characterisation of the chemical gradient across the diamond surface treated with air plasma: Position 2 (more exposed side) (a) shows the entire XPS spectrum (b) an analysis of the oxygen group peak. (c) is the area of the C1s peak

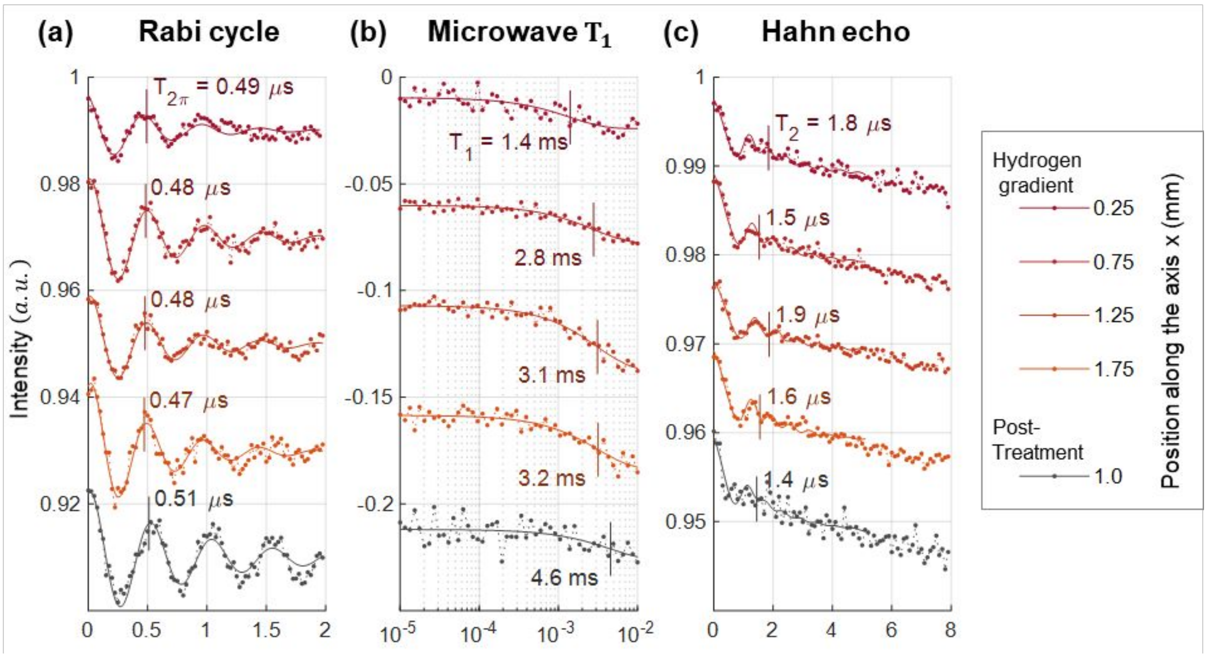

Figure S5. Rabi oscillations (a) T1 microwaves (b) and Hahn echo sequence performed in confocal configuration.

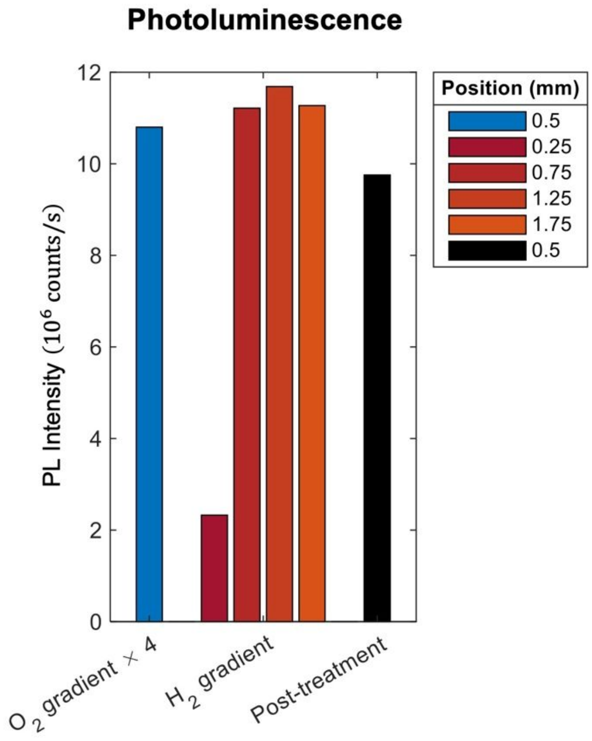

Figure S6: Photoluminensence counts from the surfaces before (blue) directly after at different positions (red) and after regeneration (black). Except for the most exposed side (0.25 mm from the open side of the mask), the count rates are relatively constant.

References

[1] Ghodbane, S., Ballutaud, D., Omnès, F. and Agnès, C., 2010. Comparison of the XPS spectra from homoepitaxial {111},{100} and polycrystalline boron-doped diamond films. *Diamond and related materials*, *19*(5-6), pp.630-636.

[2] Yang, L., Jiang, C., Guo, S., Zhang, L., Gao, J., Peng, J., Hu, T. and Wang, L., 2016. Novel diamond films synthesis strategy: methanol and argon atmosphere by microwave plasma CVD method without hydrogen. *Nanoscale Research Letters*, *11*, pp.1-6.
